# Efficacy and safety of tranexamic acid usage in patients undergoing posterior lumbar fusion: a meta-analysis

**DOI:** 10.1186/s12891-019-2762-2

**Published:** 2019-08-31

**Authors:** Jianzhong Bai, Pei Zhang, Yuan Liang, Jingcheng Wang, Yongxiang Wang

**Affiliations:** 1 Department of Orthopedics, Clinical Medical College of Yangzhou University, Northern Jiangsu People’s Hospital, Nantong West Road 98, Yangzhou, 225001 China; 2 Dalian Medical University, Dalian, 116044 Liaoning China

**Keywords:** Tranexamic acid, Posterior lumbar fusion, Blood loss, Lumbar degenerative disease, Spinal surgery

## Abstract

**Background:**

The purpose of this meta-analysis is to evaluate the efficacy and safety of tranexamic acid (TXA) for patients with degenerative lumbar disc herniation, stenosis or instability undergoing posterior lumbar fusion (PLF) surgery.

**Methods:**

We searched PubMed, Embase, and Cochrane Library until May 1, 2018. Two reviewers selected studies, assessed quality, extracted data, and evaluated the risk of bias independently. Weighted mean difference (WMD) and relative risk (RR) were calculated as the summary statistics for continuous data and dichotomous data, respectively. We chose fixed-effects or random-effects models based on I^2^ statistics. RevMan 5.0 and STATA 14.0 software were used for data analysis.

**Results:**

Nine studies enrolling 713 patients for the study. The pooled outcomes demonstrated that TXA can decrease total blood loss (TBL) in patients underwent PLF surgery [WMD = -250.68, 95% CI (− 325.06, − 176.29), *P*<0.001], intraoperative blood loss (IBL) [WMD = -72.57, 95% CI (− 103.94, − 41.20), *P*<0.001], postoperative blood loss (PBL) [WMD = -127.57, 95% CI (− 149.39, − 105.75), *P*<0.001], and the loss of hemoglobin (Hb) in postoperative 24 h [WMD = -0.31, 95% CI (− 0.44, − 0.18), *P*<0.001]. However, there is no significant difference between two groups in transfusion rate [RR =0.34, 95% CI (0.09, 1.28), *P* = 0.11], and none thrombotic event was happened in the two groups.

**Conclusion:**

Our meta-analysis demonstrated that TXA can decrease the Hb loss, TBL, IBL, PBL, and without increasing the risk of thrombotic event in patients with degenerative lumbar disc herniation, stenosis or instability underwent PLF surgery. However, there was no significant difference in blood transfusion rates between the two groups.

## Background

Posterior lumbar fusion (PLF) has achieved satisfactory results in the treatment of lumbar degenerative diseases. However, this surgical procedure was associated with substantial blood loss during the perioperative setting, which increases intraoperative hypotension, invisible operative field, postoperative neural compression, infection, anemia, and other morbidities. Blood transfusion could correct anemia, but it could cause some complications, such as hemolytic reaction, electrolyte disturbances, and infectious diseases [1, 2]. Therefore, it is necessary to reduce the blood loss for patients who underwent spinal surgery [3].

TXA can block the lysine binding sites of plasminogen, plasmin, and tissue plasminogen activator to delay fibrinolysis and blood clot degradation [4, 5]. This drug has a significant hemostatic effect in obstetric surgery, hip or knee joint replacement, and coronary-artery surgery [6–8]. Previous meta-analyses have shown that TXA can significantly reduce blood loss in spinal surgery [9–12]. However, these studies included multiple types of surgical procedures, different surgical approaches, and areas. Recently, two meta-analyses demonstrated that patients treated with TXA had a significantly lower blood loss in scoliosis surgery [13, 14]. It is well known that scoliosis surgery is extremely complicated, which operation time and blood loss are far more than the PLF surgery for lumbar degenerative diseases. It is unclear whether TXA has a similar effect in PLF surgery to the scoliosis surgery. Therefore, we conducted the meta-analysis to evaluate the efficacy and safety of TXA in PLF surgery for the treatment of lumbar degenerative diseases.

## Materials and methods

This meta-analysis was performed according to the Preferred Reporting Items for Systematic Reviews and Meta-Analysis (PRISMA) statement [15].

### Literature search

We searched the PubMed, Embase, and Cochrane Library from the date of their inception to May 1, 2018, and no language limited. Additionally, the references of the included studies were manual searched to find additional studies. The following keywords were used in the database search: “Tranexamic acid”, “Posterior lumbar fusion”, “Lumbar degenerative disease”, and “Spinal surgery”.

### Study selection and eligibility criteria

Inclusion criteria: 1) control group is placebo or saline, experimental group is TXA; 2) endpoints: TBL, IBL, PBL, postoperative 24 h Hb decline, transfusion rate, and thromboembolic events; 3) study designs were randomized controlled trials (RCTs) and case-control trials (CCTs); 4) patient is older than 18 years.

Exclusion criteria: 1) other types of spinal disorders, such as scoliosis, Duchenne muscular dystrophy, spine fractures; 2) other study designs did not provide sufficient data, such as case report, review, commentary, and so on; 3) there was duplicate publication.

### Data extraction

We extracted the general characteristics from each study: author, published year, sample size, age, patient type, study design, the dose of TXA, transfusion criteria, and outcome measures. Any discrepancies were resolved following discussion. The endpoints included TBL, IBL, PBL, postoperative Hb decline, transfusion rate, thromboembolic events.

### Quality assessment

The quality of the RCTs was accessed by the 12-items scale [16]. The score of more than 7, 5–7, and less than 5 was considered as high, moderate, and low quality, respectively. The Newcastle-Ottawa Scale was used to assess the quality of non-RCTs [17]. We set the score of 0–3, 4–6, and 7–9 for low, moderate, and high quality of the study, respectively.

### Data analysis and statistical methods

We used Revman 5.0 software and Stata14.0 to make statistical analyses. Weighted mean difference (WMD) and relative risk (RR) were calculated as the summary statistics for continuous data and dichotomous data, respectively. Two-tailed *P*<0.05 was regarded as statistically significant. Statistical heterogeneity was assessed by the I^2^ statistic, and I^2^ value more than 50% indicates significant heterogeneity, less than 50% is considered acceptable. We performed subgroup analyses based on the route of administration, study designs and the quality of studies.

## Results

### Search result

**Fig. 1:**
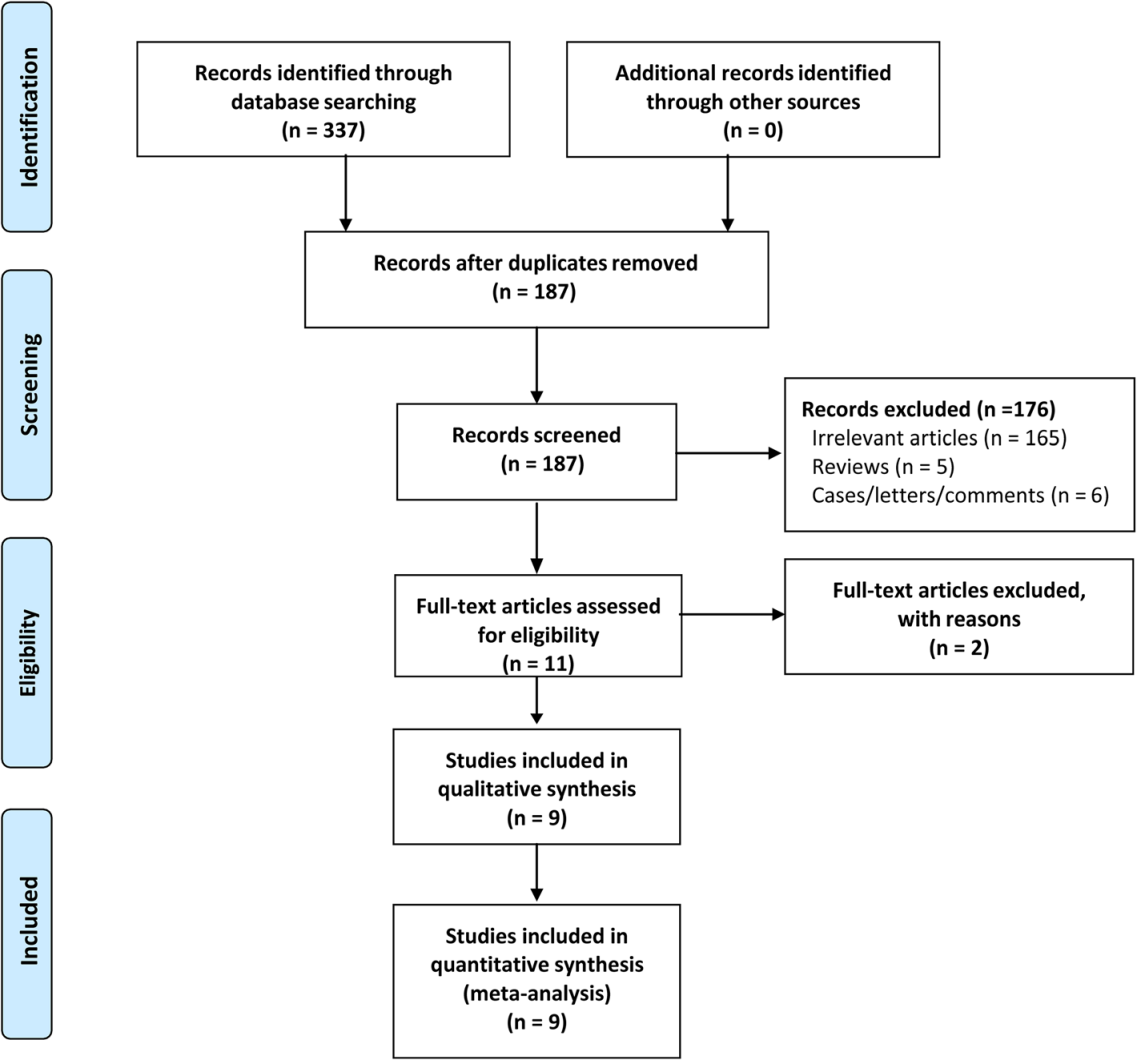
The flow chart of studies selecting

Two reviewers independently undertook the searches according to the search strategy. A total of 337 articles were retrieved, Endnote X8 (version 18.0.0.10063) was used to remove 150 duplicate studies. Additionally, we deleted 176 irrelevant articles through the title and abstract. Two studies were excluded according to full texts, which one article was duplicate publication [18] and the other did not have a suitable control group [19]. Finally, nine studies were included. The study selection process was shown in Fig. 1.

### Study characteristics

Six of nine were RCTs [20–25], and three were CCTs [26–28]. These studies were published between 2011 and 2018, with sample sizes ranging from 50 to 118 subjects, a total of 713 subjects, and the procedure with TXA was performed on 368 subjects. The main characteristics of the included trials are summarized in Table 1.

**Table 1:** Characteristics of the included studies

Study (year)	No. T VS C	Mean age (years): T VS C	Preoperative Hb (g/dl, T:C)	Study design	Patient type	Dose of TXA	Transfusion criteria	Outcome measures
Endres 2011 [28]	46/51	67/69	14.91/14.51	CCT	Lumbar degenerative spinal stenosis and instability	1 g IV preoperative, 6 h and 12 h postoperative respectively	NR	②③④
Wang 2013 [20]	30/30	63.1/62	13.7/14.1	RCT	Lumbar degenerative spinal stenosis and instability	15 mg/kg mixed in 100 ml saline before surgery	NR	①②③⑥
Kushioka 2016 [26]	30/30	67.8/71.5	13.3/13.3	CCT	Lumbar degenerative disease (single institution)	2000 mg IV, preoperative and 16 h postoperative surgery respectively	NR	①②③④⑤
Liang 2016 [21]	30/30	51.13/53.83	13.96/13.5	RCT	Lumbar degenerative spinal stenosis	2000 mg in 20 ml saline solution soaked	Hb < 7 g/dl	②③④⑤
Roopa 2017 [24]	25/25	69.0/70	NR	RCT	Lumbar degenerative disease (single institution)	10 mg/kg IV before surgery and 1 mg/kg/hr. till closure.	Hb < 8 g/dl	②③④⑥
Kim 2017 [22]	24/24/24	61/63.3/65.2	13.1/13.3/13.2	RCT	Lumbar degenerative disease (single institution)	HD group received 10 mg/kg in 100 mL of normal saline, and 2 mg/kg/h until 5 h after surgery. LD group half of the dose	NR	①②③④
Ren 2017 [27]	50/50	55.2/58.7	13.91/13.92	CCT	Lumbar disc herniation or lumbar spinal stenosis	1 g in 100 mL saline solution soaked	Hb < 7 g/dl	①②③⑥
Shi 2017 [23]	50/46	57.7/55.3	13.5/13.7	RCT	Lumbar spinal stenosis or lumbar spondylolisthesis	30 mg/kg i.v before skin incision, and a maintenance dosage of 2 mg/kg/h TA until skin closure	Hb < 7 g/dl	①②③④⑤
Ou 2018 [25]	59/59	64.2/64	12.8/12.9	RCT	Lumbar degenerative spinal stenosis and instability (double institutions)	15 mg/kg i.v after anesthesia, and 1.0 g in 10 mL of normal saline soaked	Hb < 9.0 g/dl	①②③④⑤⑥

①Total blood loss ②Intraoperative blood loss ③Postoperative blood loss ④Postoperative 24 h hemoglobin decline ⑤Transfusion rate ⑥Thromboembolic events iv: intravenous; Hb: hemoglobin; HD: high-dose; LD: low-dose; T: tranexamic acid group; C: control group; RCT: randomized controlled trial; CCT: case control trial

### Study quality

The quality of RCTs was shown in Table 2. Four studies were of high quality [20, 22–24], and two studies were of moderate quality [21, 25]. Details of the quality assessment of CCTs were shown in Table 3. The average score was 8.3 (range, 8–9), suggesting that all the studies were of high quality.

**Table 2:** The 12-item appraisal scores for the RCTs

Studies	Randomized adequately^a^	Allocation concealed	Patient blinded	Care provider blinded	Outcome assessor blinded	Acceptable drop-out rate^b^	ITT Analysis^c^	Avoided selective reporting	Similar baseline	Similar or avoided cofactor	Patient compliance	Similar timing	Quality^d^
Wang et al	Unclear	Unclear	Yes	Yes	No	Yes	Yes	Yes	Yes	Unclear	Yes	Yes	High
Liang at al	Unclear	Unclear	Unclear	Unclear	No	Yes	Yes	Yes	Yes	Unclear	Yes	Yes	Moderate
Roopa et al	Yes	Yes	Yes	Yes	Unclear	Yes	Yes	Yes	Yes	Unclear	Yes	Yes	High
Kim et al	Yes	Yes	Yes	Yes	Unclear	Yes	Yes	Yes	Yes	Unclear	Yes	Yes	High
Shi et al	Yes	Yes	Yes	Yes	Yes	Yes	Yes	Yes	Yes	Unclear	Yes	Unclear	High
Ou et al	Unclear	Unclear	Unclear	Unclear	No	Yes	Yes	Yes	Yes	Unclear	Yes	Yes	Moderate

a: Only if the method of sequence made was explicitly introduced could get a ‘Yes’

b: Drop-out rate < 20% could get a ‘Yes’, otherwise ‘No’

c: ITT = intention-to-treat, only if all randomized participants were analyzed in the group they were allocated to could receive a ‘Yes’

d: “Yes” items more than 7 means ‘High’; more than 4 but no more than 7 means ‘Moderate’; no more than 4 means ‘Low’

**Table 3:** The Newcastle-Ottawa Scale appraisal scores for the non-RCTs

Study	Selection				Comparability	Outcome			
	Definition of cases	Cases Representativeness	Selection of Controls	Definition of Controls		Assessment of Exposure	Same Methods	Non-Response Rate	Total Score
Endres et al.	*	*	*	*	*	*	*	*	8
Kushioka et al.	*	*	*	*	*	*	*	*	8
Ren et al.	*	*	*	*	**	*	*	*	9

Single asterisk indicates 1 score, double asterisk indicates 2 scores, and dash indicates 0 scores

### Clinical outcomes

#### Total blood loss (ml)

**Fig. 2:**
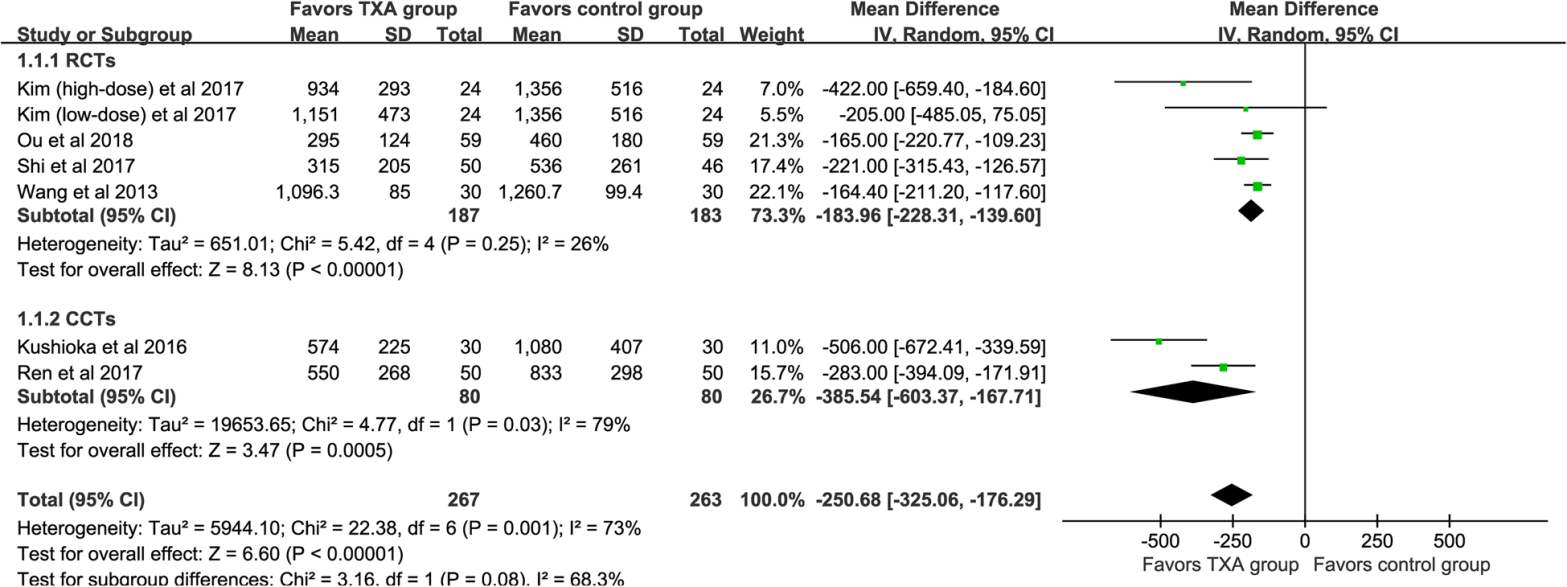
The forest plot for total blood loss

TBL was available from six studies [20, 22, 23, 25–27], and the pooled outcomes demonstrated that the TXA could significant decrease TBL [WMD = -250.68, 95% CI (− 325.06, − 176.29), *P*<0.001, I^2^ = 73%, Fig. 2].

#### Intra-operative blood loss (ml)

**Fig. 3:**
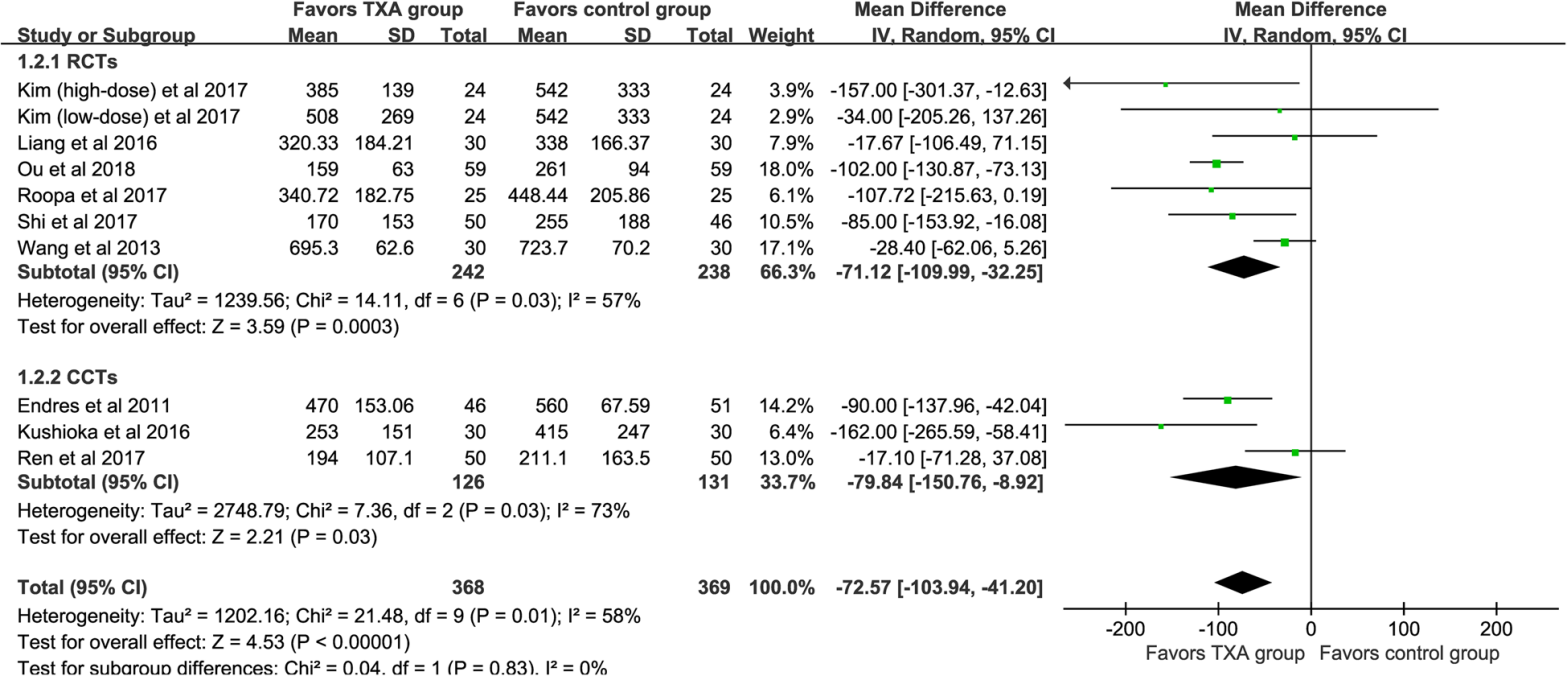
The forest plot for intraoperative blood loss

IBL was available from nine studies [20–28], and the results demonstrated that the TXA could significant decrease IBL [WMD = -72.57, 95% CI (− 103.94, − 41.20), *P*<0.001, I^2^ = 58%, Fig. 3].

#### Post-operative blood loss (ml)

**Fig. 4:**
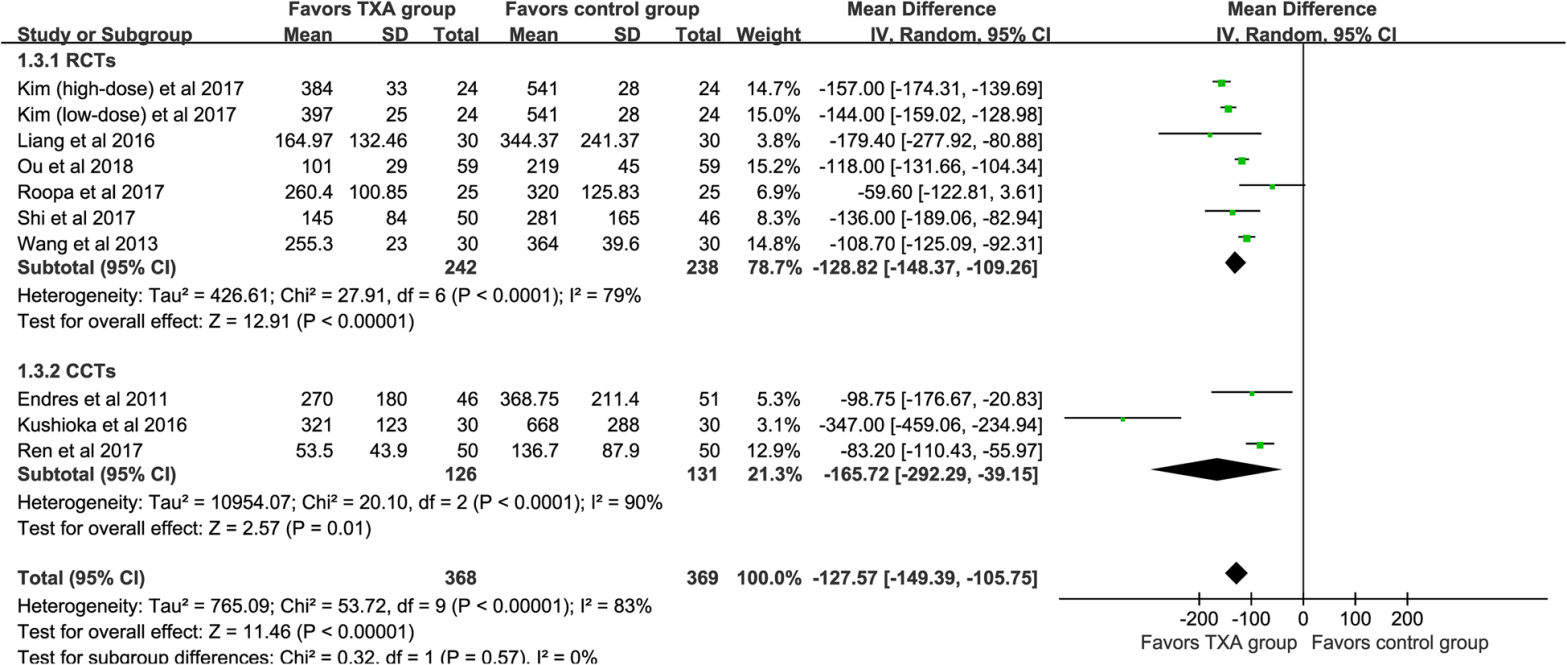
The forest plot for postoperative blood loss

PBL was available from nine studies [20–28], and the pooled ooutcomes demonstrated that the TXA could significant decrease PBL [WMD = -127.57, 95% CI (− 149.39, − 105.75), *P*<0.001, I^2^ = 83%, Fig. 4].

#### Postoperative 24 h hemoglobin decline (g/dl)

**Fig. 5:**
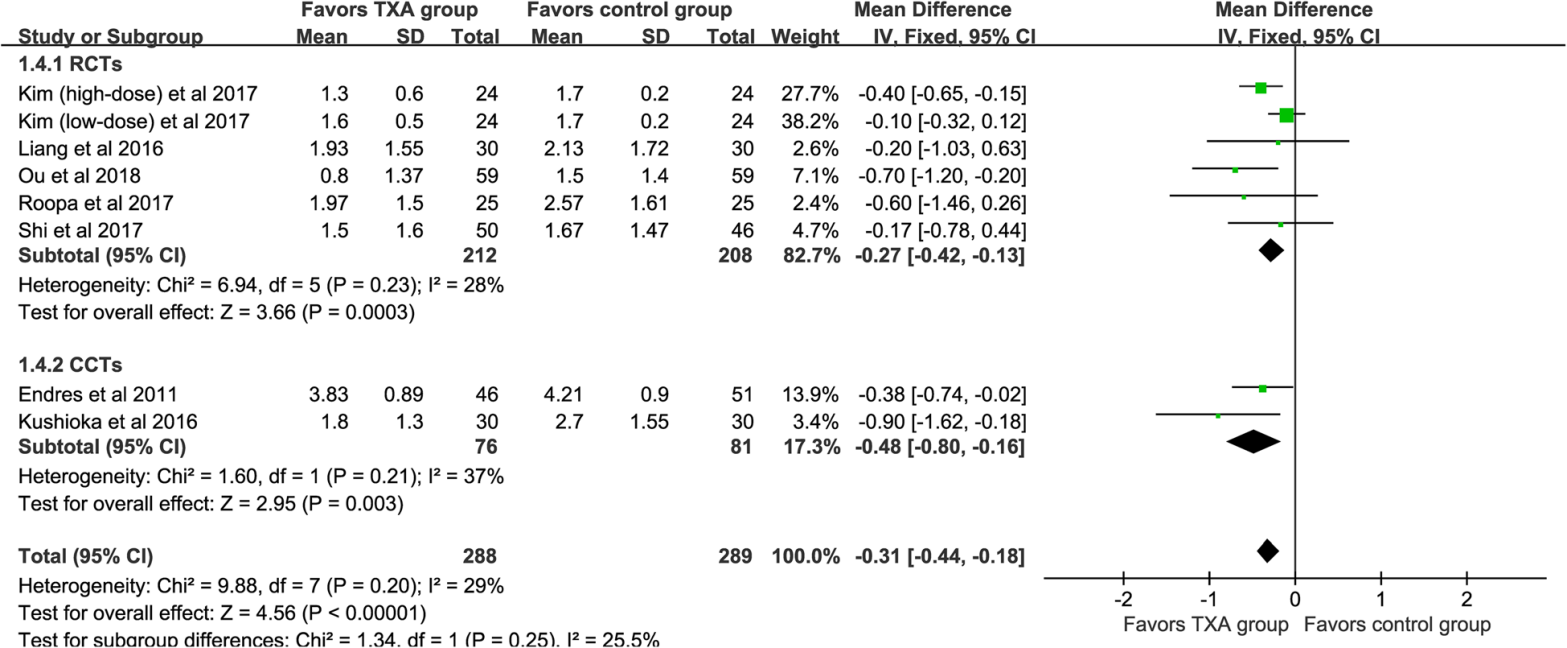
The forest plot for postoperative 24-h Hb decline value

Seven studies [21–26, 28] provided available data and the pooled outcomes demonstrated that the TXA group had a lower postoperative Hb decline value [WMD = -0.31, 95% CI (− 0.44, − 0.18), *P* < 0.001, I^2^ = 29%, Fig. 5].

#### Transfusion rate and thromboembolic events

**Fig. 6:**
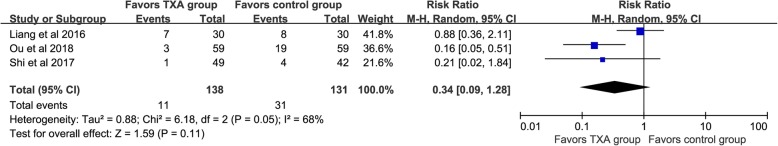
The forest plot for transfusion rate

Transfusion rate was available from three studies [21, 23, 25], and the pooled outcomes indicated that no significant difference between the two groups [RR =0.34, 95% CI (0.09, 1.28), *P* = 0.11, I^2^ = 68%, Fig. 6]. Besides, none thrombotic event was happened in the two groups [20, 24, 26–28].

### Funnel plot analysis

**Fig. 7:**
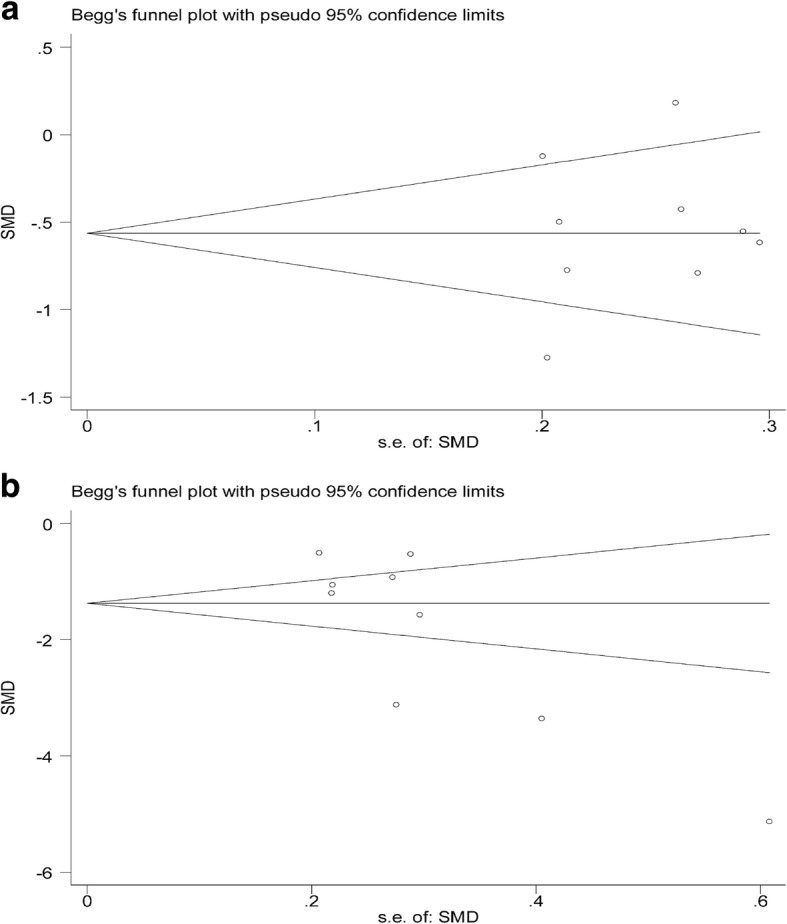
**a** The funnel plot for intraoperative blood loss. **b** The funnel plot for postoperative blood loss

IBL and PBL were used to generate the funnel plot analysis of publication bias (Fig. 7). The symmetric characteristic of the pooled plot indicated that no significant publication bias (Begg’s test: *P* = 0.917 for IBL, Fig. 7a; Begg’s test *P* = 0. 118 for PBL, Fig. 7b). Due to the limited number of studies included, so publication bias was not evaluated in other outcomes.

### Subgroup analysis

#### Different routes of administration

We conducted a subgroup analysis based on the route of administration (shown in Table 4). TBL was available from four studies [20, 22, 23, 26], and the pooled outcomes demonstrated that the intravenous TXA (IV TXA) could significant decrease TBL [WMD = -287.25, 95% CI (− 411.81, − 162.70), *P*<0.001, I^2^ = 79%]. Besides, the TBL significantly decreased in topical use of TXA (tTXA) group (*P*<0.001) [27] and combined IV with topical administration of TXA group (*P*<0.001) [25]. For IBL, six studies [20, 22–26] provided available data and the results demonstrated that the IV TXA could decrease IBL [WMD = -82.73, 95% CI (− 122.80, − 42.66), *P*<0.001, I^2^ = 48%]. Two studies [21, 27] reported available data, and the results suggested that the tTXA group could not decrease IBL significantly [WMD = -17.25, 95% CI (− 63.51, 29.00), *P* = 0.46, I^2^ = 0%]. One study [25] demonstrated that the combined IV with topical administration of TXA could significant decrease IBL (*P*<0.001). For PBL, six studies [20, 22–24, 26, 28] provided available data and the pooled results demonstrated that the IV TXA can significant decrease PBL [WMD = -135.66, 95% CI (− 164.24, − 107.08), *P*<0.001, I^2^ = 84%]. Two studies [21, 27] reported available data, and the pooled results suggested that the tTXA could significant decrease PBL [WMD = -119.17, 95% CI (− 210.40, − 27.94), *P* = 0.01, I^2^ = 71%]. One study [25] demonstrated that the combined IV with topical administration of TXA could decrease PBL (*P*<0.001). We further conducted a subgroup analysis for postoperative 24 h hemoglobin decline based on the route of administration (shown in Table 4). Five studies [22–24, 26, 28] provided available data and the results demonstrated that the IV TXA group with a lower postoperative Hb decline value [WMD = -0.28, 95% CI (− 0.42, − 0.14), *P* < 0.001, I^2^ = 32%].

**Table 4:** Subgroup analysis based on the route of administration and the study quality

Outcomes	Subgroup	Study (N)	RR/WMD	95%CI	I^2^ (%)	*P*
Total blood loss	IV TXA	4	-287.25	(−411.81,-162.70)	79	<0.001
	tTXA	1	− 283	(− 394.09,-171.91)	–	<0.001
	Combined	1	− 165	(− 220.77,-109.23)	–	<0.001
Intraoperative blood loss	IV TXA	6	−82.73	(−122.80,-42.66)	48	<0.001
	tTXA	2	−17.25	(−63.51,29.00)	0	0.46
	Combined	1	− 102	(− 130.87,-73.13)	–	<0.001
Postoperative blood loss	IV TXA	6	−135.66	(−164.24,-107.08)	84	<0.001
	tTXA	2	−119.17	(−210.40,-27.94)	71	<0.001
	Combined	1	−118	(− 131.66,-104.34)	–	<0.001
Postoperative 24 h HB decline	IV TXA	5	−0.28	(−0.42,-0.14)	32	<0.001
	tTXA	1	−0.2	(−1.03,0.63)	–	0.64
	Combined	1	−0.7	(−1.20,-0.2)	–	0.006
Total blood loss	High	4	− 208.93	(− 286.07,-131.78)	42	<0.001
	Moderate	1	−165.00	(−220.77,-109.23)	–	<0.001
Intraoperative blood loss	High	4	−64.12	(−108.32,-19.91)	31	0.004
	Moderate	2	−70.73	(−150.56,9.11)	68	0.08
Postoperative blood loss	High	4	−128.51	(−153.93,-103.08)	83	<0.001
	Moderate	2	−128.52	(− 173.86,-83.18)	32	<0.001
Postoperative 24 h HB decline	High	3	−0.23	(−0.39,-0.08)	23	0.003
	Moderate	2	−0.57	(−0.99,-0.14)	3	0.009

IV: intravenous use of TXA; tTXA: topical use of TXA; Combined: combined IV administration and topical application of TXA; −: no available; High: high quality; Moderate: moderate quality

#### Different study designs

We divided the included studies into RCTs and CCTs, and subgroup analysis based on different designs of the studies. For TBL (shown in Fig. 2), four RCTs demonstrated that the TXA could decrease TBL [WMD = -183.96, 95% CI (− 228.31, − 139.60), *P*<0.001, I^2^ = 26%]. The results of the pooled of the two CCTs were similar to the former [WMD = -385.54, 95% CI (− 603.37, − 167.71), *P*<0.001, I^2^ = 79%]. Six RCTs and three CCTs provide available data, respectively, and the pooled results showed that TXA could significantly reduce the IBL (*P*<0.05, shown in Fig. 3). Similarly, both types of studies have shown that TXA can significantly reduce the PBL in patients (*P*<0.05, shown in Fig. 4). Five RCTs and two CCTs provide available data, and the results showed that TXA group had a lower postoperative Hb decline value (*P*<0.05, shown in Fig. 5).

#### Studies with different quality

The quality score of the CCTs was more than 7 points and considered as high-quality studies, so no subgroup analysis was performed. For RCTs, the pooled results of the high-quality studies showed that TXA group had a significant decrease in TBL, IBL, PBL, and the loss of Hb than the control group (*P*<0.05, shown in Table 4). For RCTs with moderate quality, the pooled outcomes demonstrated that the TXA group has less blood loss than the control group, similar to high-quality RCTs (shown in Table 4), but there is no significant difference between the two groups in IBL (*P* = 0.08, shown in Table 4), which may be caused by the limitation of studies.

### Sensitivity analysis

Due to the limited number of studies in tTXA and combined IV with topical administration, we only conducted sensitivity analysis in the data of IV TXA. The exclusion of each study once a time in TBL, IBL, PBL, and postoperative 24 h HB decline, and all the conclusions kept stable.

## Discussion

The main findings of this meta-analysis demonstrated that TXA could decrease the postoperative Hb loss, TBL, IBL, PBL, and without increasing the risk of the thrombotic event in PLF surgery. However, there is no statistical difference in transfusion rate between the two groups. The results of our meta-analysis demonstrated that the hemostasis effect of TXA in PLF surgery was similar to scoliosis surgery [14].

TXA has been widely used in clinical treatment as a hemostatic agent, and it was included on the WHO list as an essential medicine in 2011 [29]. The safety of TXA has been confirmed in obstetric surgery, hip and knee joint replacement, coronary-artery and other surgeries [6–8]. The study of Benoni et al. pointed out that TXA does not increase the risk of DVT, and it just inhibits fibrinolysis in the wound to play a hemostasis effect [30]. A large sample size study involving 872,416 patients demonstrated that TXA does not increase the risk of postoperative complications [31]. No thromboembolic events were found in TXA groups as well in our meta-analysis. However, due to the lack of literature and the different route of administration, the conclusion of transfusion rate is not convincing.

IBL directly affects the operation time and visible surgical field, so it is of considerable significance to the surgery. However, it is unclear whether tTXA can significantly reduce IBL in PLF surgery. The study of Ren et al. indicated that tTXA had a similar effect to the control group in PLF surgery [27]. But Liang et al. point out that TXA soaked gelfoam group had a similar amount of blood loss compared with gelfoam group during the PLF surgery [21]. tTXA was mainly absorbed into the blood to exert the hemostatic effect. Whether tTXA could quickly exert hemostatic effect to decrease IBL during PLF surgery should be taken into consideration.

It is unclear whether the effect of combined IV administration with topical application of TXA is superior to single-use of TXA. The meta-analysis of Sun et al. [32] demonstrated that IV combine with tTXA was better than IV TXA in total hip replacement regarding blood loss, hemoglobin decline, and transfusion rate. Yang et al’s study [33] indicated that the combined administration of TXA in total joint arthroplasty was superior to the single use of TXA. Ou et al’s study [25] suggested that combined IV administration with topical application of TXA could significantly decrease allogeneic blood transfusion and blood loss in PLF surgery. Therefore, it is of some significance to consider this method in spinal surgery.

Currently, there was no guideline to specify the dosage of TXA in spine surgery. Kim et al’s study [22] suggested that high-dose TXA (10 mg/kg of bolus loading dose and 2 mg/kg/h of continuous infusion) was more effective than low-dose TXA in the decrease of blood loss in PLF surgery. Xie et al’s study [34] suggested that high-dose TXA (100 mg/Kg of bolus loading dose and 10 mg/Kg/h until skin closure) could effectively control blood loss and reduce the transfusion requirement without adverse drug reactions in spine correction surgery. The study of Kushioka [26] demonstrated that high-dose TXA (2000 mg) decreased both IBL and PBL without causing any side effects in PLF surgery. A recent meta-analysis suggested that high-dose TXA has a better hemostatic effect than the control group in patients underwent PLF surgery [35], but the dose of TXA administered during and after the operation is different in the included studies, so the conclusion needs further discussion. Lin et al. [36] pointed out high-dose (50 mg/kg IV loading dose followed by a 5-mg/kg/h infusion until skin closure) was efficacy in complex adult spinal deformity surgery, but there were three thromboembolic postoperative complications. The dosages of TXA should be determined according to the type of spinal surgery, the patient’s conditions (e.g., weight, renal function) and other factors. Besides, the relevant dose-response analysis should be performed to identify the optimal TXA dose in spinal surgery.

We recommend future research as follows. (1) The most appropriate time and dosage for TXA need further study. (2) Topical TXA is considered to have less influence on the systemic fibrinolytic system and is safer than IV TXA, but its efficacy requires further research. (3) It is unclear whether the use of combined IV administration and topical application of TXA is superior to IV in spinal surgery. (4) For spinal surgery, PBL may increase the formation of epidural hematomas that cause neurological disorders [37]. Combined with Table 1 and Fig. 4, we can see that postoperative TXA can significantly reduce the amount of PBL in patients, so whether TXA is needed after surgery for patients with suspected bleeding should be made the further study. (5) Most of the previous studies have excluded patients with severe heart and lung diseases, however, for these patients, reducing blood loss in perioperative blood loss is truly meaningful, so relevant research should be further exploring the use of TXA in these type patients in spinal surgery. (6) It has been reported that oral administration of TXA had comparable hemostasis effect to IV TXA and topical TXA in total hip/knee arthroplasty [38], and it has been found with higher safety level. Therefore, it is significant to consider oral TXA in spinal surgery.

Limitations are as follows. (1) Due to the lack of guidelines for clinical use of TXA, the doses and timing of TXA usage are unequal in the included studies. It was impossible to perform sub-analysis of them to make a further explanation. (2) Some pooled outcomes have higher heterogeneity, different time and the dose of tranexamic acid used, and diverse transfusion criteria may be contributed to it. (3) Most studies included in this meta-analysis were small sample size and some non-RCTs, which may affect the reliability of the conclusions.

## Conclusion

Our meta-analysis demonstrated that TXA can decrease the Hb loss, TBL, IBL, PBL, and without increasing the risk of thrombotic event in patients with degenerative lumbar disc herniation, stenosis or instability underwent PLF surgery. However, there was no significant difference in blood transfusion rates between the two groups.

## Data Availability

All data are fully available without restriction.

## Abbreviations

CCTs: Case control trials
CI: Confidence intervals
Hb: Hemoglobin
I^2^: I-square
IBL: Intraoperative blood loss
IV: Intravenous use of TXA
PBL: Postoperative blood loss
PLF: Posterior lumbar fusion
RCTs: Randomized controlled trials
RR: Relative risk
TBL: Total blood loss
tTXA: topical use of TXA
TXA: Tranexamic acid
WMD: Weighted mean difference
